# From Mouse to Man and Back: Closing the Correlation Gap between Imaging and Histopathology for Lung Diseases

**DOI:** 10.3390/diagnostics10090636

**Published:** 2020-08-26

**Authors:** Birger Tielemans, Kaat Dekoster, Stijn E. Verleden, Stefan Sawall, Bartosz Leszczyński, Kjell Laperre, Arno Vanstapel, Johny Verschakelen, Marc Kachelriess, Erik Verbeken, Jim Swoger, Greetje Vande Velde

**Affiliations:** 1 Department of Imaging and Pathology, KU Leuven, University of Leuven, 3000 Leuven, Belgium; birger.tielemans@kuleuven.be (B.T.); kaat.dekoster@kuleuven.be (K.D.); johny.verschakelen@kuleuven.be (J.V.); erik.verbeken@kuleuven.be (E.V.); 2 Department of CHROMETA, BREATHE lab, KU Leuven, 3000 Leuven, Belgium; stijn.verleden@kuleuven.be (S.E.V.); arno.vanstapel@kuleuven.be (A.V.); 3 German Cancer Research Center (DKFZ), X-Ray Imaging and CT, Heidelberg University, 69117 Heidelberg, Germany; stefan.sawall@dkfz-heidelberg.de (S.S.); marc.kachelriess@dkfz-heidelberg.de (M.K.); 4 Department of Medical Physics, M. Smoluchowski Institute of Physics, Faculty of Physics, Astronomy and Applied Computer Science, Jagiellonian University, 31-007 Kraków, Poland; bartosz.leszczynski@uj.edu.pl; 5 Bruker Belgium, 2550 Kontich, Belgium; Kjell.Laperre@bruker.com; 6 European Molecular Biology Laboratory (EMBL) Barcelona, 08003 Barcelona, Spain; swoger@embl.es

**Keywords:** imaging-histopathology correlation, μCT, MRI, optical imaging, virtual biopsy, clinical, experimental, lung fibrosis

## Abstract

Lung diseases such as fibrosis, asthma, cystic fibrosis, infection and cancer are life-threatening conditions that slowly deteriorate quality of life and for which our diagnostic power is high, but our knowledge on etiology and/or effective treatment options still contains important gaps. In the context of day-to-day practice, clinical and preclinical studies, clinicians and basic researchers team up and continuously strive to increase insights into lung disease progression, diagnostic and treatment options. To unravel disease processes and to test novel therapeutic approaches, investigators typically rely on end-stage procedures such as serum analysis, cyto-/chemokine profiles and selective tissue histology from animal models. These techniques are useful but provide only a snapshot of disease processes that are essentially dynamic in time and space. Technology allowing evaluation of live animals repeatedly is indispensable to gain a better insight into the dynamics of lung disease progression and treatment effects. Computed tomography (CT) is a clinical diagnostic imaging technique that can have enormous benefits in a research context too. Yet, the implementation of imaging techniques in laboratories lags behind. In this review we want to showcase the integrated approaches and novel developments in imaging, lung functional testing and pathological techniques that are used to assess, diagnose, quantify and treat lung disease and that may be employed in research on patients and animals. Imaging approaches result in often novel anatomical and functional biomarkers, resulting in many advantages, such as better insight in disease progression and a reduction in the numbers of animals necessary. We here showcase integrated assessment of lung disease with imaging and histopathological technologies, applied to the example of lung fibrosis. Better integration of clinical and preclinical imaging technologies with pathology will ultimately result in improved clinical translation of (therapy) study results.

## 1. Current Challenges in Lung Disease Management

Computed tomography (CT) is an integral part of the diagnostic and therapeutic workup of many lung diseases. This imaging technique produces images that can be used for diagnosis, to determine therapeutic options and to follow-up on the outcome of the patient during treatment. Unfortunately, treatment is often limited to relieving symptoms or stopping progression since curative treatment is not always possible or available. Lung diseases are among the most important causes of illness and death in the world nowadays. Chronic obstructive pulmonary disease, lung cancer, fibrosis, emphysema and asthma are examples of such devastating and life-threatening conditions. Researchers are therefore continuously investigating novel strategies to prevent and cure lung diseases. Even when sometimes the cause is obvious (e.g., cigarette smoking, allergens, environmental pollutants), many aspects of what exactly causes the disease remain to be understood in order to find targets for new, effective treatment options.

Whereas imaging examinations are routine clinical practice, preclinical investigators typically rely on the analysis of lung tissue samples from indispensable animal models (mainly rodents) to unravel disease processes in the lung. Although these and other end-stage techniques provide plenty of opportunities for detailed molecular and microscopic evaluation of pathology, they are limited to one measurement per animal, providing only a snapshot of processes that are essentially dynamic. The identification of important factors for disease onset and progression and putative therapies are often investigated in survival studies, comparing delays in death between experimental and control groups. Animals are left to succumb to disease, while every opportunity to investigate what happened during disease progression within the lungs—pathology and host response thereto—is lost. 

At the same time, we are confronted with important challenges related to the translation of mouse study results to patients. Regarding the treatment of, for example, lung fibrosis, most if not all of the putative therapies that were successfully tested in rodent models afterwards failed in clinical studies [1,2]. Failure to translate “proof-of-principle” approaches from in vitro and in vivo models has discouraged scientists, clinicians and pharmaceutical companies in the management of fibrotic disorders such as idiopathic lung fibrosis or fibrosis in the context of systemic sclerosis or environmental exposure [1,3]. For these devastating conditions, effective anti-fibrotic treatment is still lacking [4].

Specific technological advances and optimization of existing approaches should now answer to the so-called translational challenges in the field. We put forward that combined quantitative in vivo assessment of the functional and structural changes throughout disease progression in each individual animal should increasingly be used to overcome challenges in translation of laboratory research to clinical applications. Improved quantitative and dynamic imaging of animal models should increase knowledge of the kinetics of pathophysiological processes at play. More accurate description of the effects of therapeutic interventions will increase the probability of successful translation to clinical practice, ultimately reducing unnecessary financial expenses or animal use and improving target selection for clinical trials. 

Imaging of small animal models of disease provides unique opportunities for model evaluation and for obtaining mechanistic insights into pathological processes. Micro-computed tomography (µCT) in particular, and also magnetic resonance imaging (MRI), provide specific insights into the onset and progression of fibrotic lung disease and response to treatment [4,5,6,7,8,9]. These approaches overcome several limitations that were previously associated with animal models of fibrosis: most scoring methods relied on single end-point measurements of what is essentially a dynamic disease process in an individual animal. 

Yet, despite the validation and the obvious advantages of quantitative imaging outcomes, the semi-quantitative assessment through a scoring system by histological analysis of isolated organs post mortem remains the current and often only gold standard. Whereas ex vivo analyses of tissue samples can provide the most detailed cellular and molecular analysis of the disease, they are intrinsically unable to follow the kinetics of disease and host response processes which are, by definition, dynamic in space and time. Ex vivo analyses require multiple animals to be sacrificed at several time points during the study, carrying the ethical burden of overconsumption of animals to overcome the statistical issues inherent to high inter-animal variation that would otherwise reduce the power of experiments. Moreover, one- or two-dimensional sampling alone is too limited for processes that are not evenly distributed throughout an entire organ, such as the pulmonary lobes in lung fibrosis [6]. In addition, the sensitivity, specificity and available readouts associated with non-invasive pulmonary function measurements indicate the considerable limitations of ex vivo approaches [10,11,12]. They are valuable options and provide a lot of very relevant and meaningful information, but these should be regarded as end-stage measurements. Moreover, they are limited in providing information on the spatial distribution of pathology, obscuring possible heterogeneity in disease manifestation and distribution throughout the lungs. 

New technologies are indispensable to provide, in a non-invasive manner, multiple relevant morphological and functional biomarkers to longitudinally describe how the lungs change during disease and therapy in animal models of lung disease.

Because we rely entirely on investigations using experimental animal models in order to close the gaps in our knowledge regarding lung diseases and treatment, the accurate evaluation of what happens in individual animal models of disease and the correlation between different, often complementary readouts are of crucial importance for the interpretation and interpolation towards the clinical context. Because the current gold standard methodology that is used in lung research labs is not able to capture the dynamic nature of disease processes and host responses, we believe that the lung research field would benefit from a technological leap forward from the current methodological paradigm. 

Our aim is to here provide an overview of what the current available diagnostic imaging technologies have to offer towards gaining new insights that would bridge the current translational gap and to push forward our knowledge regarding lung disease etiology and progression, with the ultimate aim of identifying new therapeutic targets and strategies. Our specific aim for this review is to improve the current standard workflow by demonstrating the added value of novel technologies that can contribute to better mechanistic knowledge of lung disease processes in the context of lung fibrosis such as parenchymal fibrosis and airway remodeling, its relation with inflammation and potential treatment strategies, with the ambition of improving lung disease management in lung fibrosis and beyond. 

## 2. Imaging and Pathology for Diagnosis of Lung Fibrosis in Clinical Practice

### 2.1. Diagnostic Imaging of ILD in Clinical Practice

High resolution CT (HRCT) is the major component in the differential diagnosis and follow-up of interstitial lung diseases (ILD). Other imaging modalities such as MRI are currently not routinely used in clinical examinations. ILD encompasses a group of more than 200 lung diseases of which idiopathic pulmonary fibrosis (IPF) is the most frequent. A multidisciplinary expert team consisting amongst others of pneumologists, radiologists and pathologists is mandatory for the diagnosis of IPF of which diagnosis is based on combining CT findings [13] with clinical history, pulmonary function testing, serological testing, bronchoalveolar lavage (BAL) analysis and, when necessary, histopathology. The use of HRCT has reduced the use of lung biopsy to confirm diagnosis of IPF. Therefore, lung biopsies are currently only recommended for those cases where major discordancy is seen between CT and clinical findings [13].

### 2.2. HRCT-Derived Biomarkers of UIP

**Figure 1:**
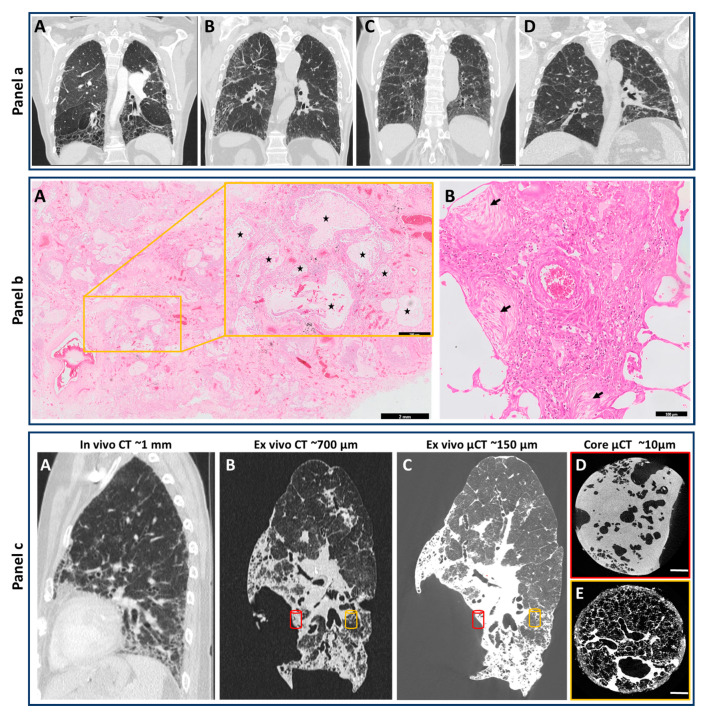
HRCT and Histopathological features for diagnosis of human UIP pattern, and overview of the potential use of CT and µCT for understanding the clinical pathology of end-stage IPF. Panel (**a**), HRCT: (**A**) Typical UIP in the setting of IPF. The presence of a peripheral, subpleural predominantly basal irregular reticular pattern, basal honeycombing and peripheral traction bronchiectasis makes IPF very likely; (**B**) Probable UIP in the setting of IPF. Peripheral and subpleural irregular reticular pattern with distal traction bronchiectasis but without honeycombing makes IPF very likely in case also clinical findings suggest this diagnosis; (**C**) Nonspecific interstitial pneumonia (NSIP). The dominant presence of ground-glass opacity together with a fine regular reticular pattern and proximal traction bronchiectasis makes IPF less likely; (**D**) Chronic fibrotic hypersensitivity pneumonitis. Irregular lines and reticular pattern are present throughout both lungs without peripheral dominance. In addition, lung density is inhomogeneous. IPF is unlikely and clinical arguments for an alternative diagnosis should be looked for. Panel (**b**): Histopathology of human lung (HE-staining). (**A**) Presence of honeycombing (inset, enlarged from yellow box, black stars) by formation of cystic airspaces of varying sizes filled with mucus and lined by bronchiolar epithelium (A scale bar = 2 mm, scale bar in inset = 500 µm); (B) Fibroblast foci showing interstitial ongoing fibrosis without inflammatory infiltrates are indicated by black arrows (scale bar = 100 µm). Panel (**c**): In vivo chest HRCT scan 6 months prior to transplantation with a resolution of around 1 mm (**A**); Ex vivo CT of the transplanted lung highlighting an increased spatial resolution due to the absence of breathing artefacts (**B**); Whole lung ex vivo µCT showing sagittal view with a resolution up to 150 µm (**C**); Core µCT with a resolution of 10 µm provides insight into different areas within the lower lobe showing severe fibrosis (red cylinder) and a near-normal area (orange cylinder) demonstrating the need for rigorous characterization of separate regions within the same lung specimen (**D**,**E**).

The HRCT correlate of IPF is usual interstitial pneumonia (UIP). HRCT features seen in UIP typically include irregular reticulation, honeycombing, traction bronchiectasis and traction bronchiolectasis with the potential presence of ground-glass opacification, fine reticulation and pleural thickening [14,15] (Figure 1a). Honeycombing refers to clusters of cystic airspaces of consistent diameter and presents as multiple layers of subpleural cysts on top of each other. Subpleural and basal presence of traction bronchiectasis/bronchiolectasis is a key feature of pulmonary fibrosis in the setting of IPF, especially when associated with the presence of honeycomb cysts [16,17]. Traction bronchiectasis/ bronchiolectasis and honeycombing are however not always present, making diagnosis of IPF more difficult. In addition, UIP is also seen in other lung diseases like hypersensitivity pneumonitis, drug-induced lung disease and some collagen vascular diseases and therefore require a multidisciplinary discussion. Ground-glass opacification, which presents as a region of slightly increased lung density in which the edges of the pulmonary vessels and airways stay visible [14], is not a typical feature of IPF but can be seen in early disease.

### 2.3. Histopathological Features of the UIP Pattern

The pathological correlate of IPF is UIP. However, no pathognomonic histological criteria exist for the diagnosis of UIP, and the diagnosis is based on detection of several major histological criteria [13] (Figure 1b). Dense fibrosis with hyalinized collagen is typically found destructing the alveolar architecture, and presents with patchy involvement, most pronounced in the subpleural and paraseptal area. Normally preserved lung parenchyma may persist in the centrilobular regions. In the fibrotic zones, an accompanying mild inflammatory infiltrate may be present. Honeycomb changes are another major finding, characterized by formation of cystic airspaces of varying size filled with mucus, and lined by bronchiolar epithelium [13]. Fibroblast foci, indicating active fibrogenesis, are often present and characterized by proliferation of myofibroblasts with a pale matrix, directly adjacent to advanced fibrotic regions. In addition, many other histological findings may be present (e.g., peribronchiolar metaplasia, emphysema). Four levels of certainty for the histological diagnosis of UIP have been proposed. A definite diagnosis of UIP can only be made when there is presence of marked fibrosis with architectural destruction, patchy involvement and fibroblast foci [18]. In contrast, presence of granulomas, marked inflammation outside fibrotic regions, hyaline membranes, preferential airway-centered involvement and organizing pneumonia, are not consistent with the diagnosis of a UIP pattern, and point towards an alternative diagnosis.

### 2.4. BAL Analysis in Parallel with HRCT

In the evaluation of patients with suspected ILD, BAL held considerable promise to diagnose and identify various subtypes of ILD. BAL is used to assess the micro-environment of the airways and is able to gain further insight in the molecular processes present within the lung, but is a relatively invasive procedure. Nowadays, the advancements in HRCT and non-invasive detection of specific scanning patterns allow differentiation of certain forms of ILD and reduce the clinical utility of BAL [19]. However, BAL cellular characterization is still a useful tool in conjunction with radiological examination to confirm and fine-tune the clinical diagnosis, which results in improved patient management [20] and is also considered an important research tool. For example, analysis of the bacterial burden in BAL fluid show this to be a risk factor for disease progression and mortality, independent of the results assessed by longitudinal HRCT [21].

### 2.5. The Role of HRCT in Clinical Lung Fibrosis Research

Accurate assessment of disease severity and progression over time is crucial in estimating clinical prognosis and efficacy of potential therapeutics. Currently, the standard primary end-point in clinical trials towards IPF is a change in forced vital capacity (FVC) to monitor disease progression. Obtaining a sensitive and objective biomarker that is able to accurately assess disease progression is lacking. Despite the essential role of HRCT in the evaluation of patients with pulmonary fibrosis, it is only used as entry criteria into clinical trials due to inter-observer variation and appears to be insufficient for precise longitudinal evaluation [22]. Due to this inter-observer variability, the prognostic information by visual assessment of scanning patterns appears inaccurate. Advances in HRCT image analysis such as observer-independent and deep-learning approaches hold a strong potential towards improving this in the future [23,24]. 

Given the aforementioned incremental importance of imaging in diagnosis and management of pulmonary fibrosis, a lot of research has focused on more extensive in vivo and ex vivo use of imaging. Initially, this was mostly centered on visual scoring by a radiologist, where the extent of fibrosis or visible honeycombing was associated with mortality [25]. More recently, the change in traction bronchiectasis was also demonstrated to be of prognostic importance, and proved to be more predictive of mortality than FVC [26]. There is, however, a strong inter-observer variability in estimating the extent of these alterations, limiting its universal applicability [27]. This has led to the development of automated CT analysis in the field of pulmonary fibrosis. First, simple measures of lung density were used, but this later evolved to more complicated algorithms. CALIPER is such a well-validated software using pathological confirmed imaging data that it can accurately predict mortality [28]. This software was also able to define a new pattern, namely vessel related structures, which was a strong predictor of outcome [29]. In addition to delivering very important clinical information, ex vivo CT has also advanced our knowledge on the pathophysiology of pulmonary fibrosis, especially when combined with corresponding µCT (Figure 1c). Indeed, especially those areas containing no or limited fibrosis in CT scans are of particular interest, as these can provide some clues about the early pathophysiological mechanisms. Those areas without fibrosis on CT show small areas of increased attenuation on µCT, possibly reflecting fibroblast foci [30]. Interestingly, small airways were equally lost in samples characterized as minimally fibrotic or severely fibrotic on CT, indicating that small airway loss could also be an important process in restrictive lung disease [31]. The implementation of frozen µCT has advanced the field even more, as structural and morphological information derived from µCT could be combined with biological studies [32]. Using µCT to define areas of minimal, moderate and severe fibrosis, Mcdonough et al. demonstrated that a core set of genes increasing or decreasing expression before fibrosis was histologically evident, and that this genetic signature continued to change with advanced fibrosis [33].

### 2.6. MRI of the Lung in Clinical Practice

The role of MRI in imaging of the heart and the great vessels is well established. For many years MRI was also a valuable “problem solving” technique in addition to CT for imaging the mediastinum, chest wall, diaphragm and lung. Nowadays however, and this is in a major part related to its technical improvement, CT is, together with chest X-ray, the major thoracic imaging modality to examine the chest including the areas thought previously to be the domain of problem-solving MRI. However, in patients who cannot receive intravascular iodine or in case radiation should be avoided, MRI of the chest may be considered as an alternative examination. Nevertheless, many research and development efforts have been made during recent years which have resulted in new and valuable applications that are very promising and that could once be implemented in clinical practice. There has been much interest in the role of MRI in the diagnosis of pulmonary embolism as a radiation-free alternative to CT [34]. In addition, imaging of pulmonary ventilation by MRI has become possible [35]. Diffusion-weighted magnetic resonance is another interesting application which has been used successfully to differentiate between malignant and benign lesions [36].

## 3. The Multimodal Toolbox for Biomedical Lung Disease Research

### 3.1. Correlating Non-Invasive In Vivo Imaging with Non-Destructive Ex Vivo Organ Imaging and Histopathology

Imaging modalities like µCT, MRI and optical imaging are indispensable and able to provide, in a non-invasive manner, a number of relevant morphological and functional measures to describe how different aspects of organs such as the lung change during health, disease and therapy in live animal models of lung disease [4,37]. In combination with one- and two-dimensional sampling methods, they result in an innovative technological pipeline based on combined imaging and lung function measurements that provide anatomical and functional biomarkers of lung disease and therapy dynamics in preclinical rodent models and whole-organ samples, up to detailed 2D examinations of isolated tissue. 

We set out to illustrate the capabilities of different in vivo and ex vivo imaging modalities in animal models, in order to unravel different aspects of lung diseases, such as inflammatory processes versus tissue/airway remodeling. This will allow identification of onset and early changes in the inflammatory versus remodeling phase, enabling, for example, full evaluation of specific antifibrotic therapeutics. 

### 3.2. Non-Invasive Modalities for Longitudinal In Vivo Lung Imaging

Importantly, introducing a longitudinal imaging approach in standard preclinical research practice reduces multifold the number of animals and animal suffering involved in longitudinal assessment of disease development and subsequent therapy testing, thereby improving the power of experiments, significantly reducing the large ethical burden associated with animal experimentation, and improving translatability of results. Moreover, the possibility to perform longitudinal studies, reducing variability in the analysis by quantitative parameters and increasing research throughput by automating image processing, are bound to provide long-term savings from the reductions in the number of animals and person-hours required for experimental work and analysis. Moreover, several imaging-derived biomarkers for lung disorders are fully translational and enable the setup of scientific collaborations and a service platform for the preclinical evaluation of innovative (antifibrotic) therapeutics. 

#### 3.2.1. In Vivo Longitudinal Lung µCT and µCT-Derived Biomarkers

**Figure 2:**
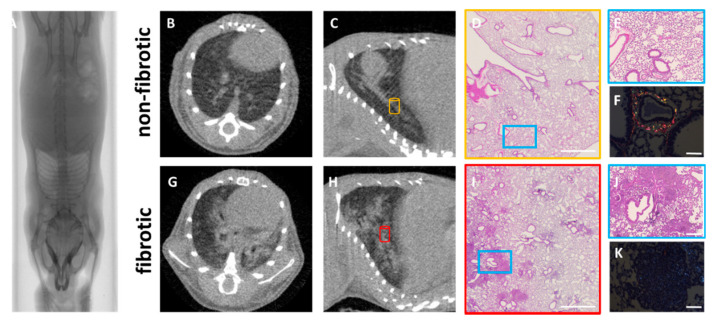
Overview of the use of µCT and histology in a pre-clinical setting in a silica-induced lung fibrosis mouse model. Mice were oropharyngeally instilled with crystalline silica particles (5 mg/mouse, bottom row) or saline (top row). Whole-body µCT scan showing the lungs of a mouse instilled with silica to induce fibrosis (**A**). Axial (**B**,**G**) and sagittal (**C**,**H**) views of non-fibrotic and fibrotic lungs. Lung fibrosis is confirmed using histology at low magnification (12.5×—scale bar 2 mm) (**D**,**I**) and high magnification (50×–scale bar 0.5 mm—blue box) from areas indicated with yellow and red cylinders in (**C**,**H)**, showing normal and dense fibrotic regions consisting of silica clustering, cellular infiltration and granuloma formation (**E**,**J**). Polarization microscopy (scale bar 0.1 mm) (**F**,**K**) allows visualization and quantification of the maturity of fibrotic lesions and silica clustering.

µCT has an enormous yet unexploited potential for preclinical lung research. This potential has already been demonstrated for different lung diseases e.g., emphysema, fibrosis, infection, cancer and metastasis to longitudinally follow up disease processes [6,7,9,38,39,40]. Similar to the clinical setting, µCT offers the capability to visually examine the entire lung for the presence of pathological alterations, evidenced by the appearance of radiodense areas that could indicate the presence of inflammatory infiltrates, lung consolidations, up to atelectatic or fibrotic areas in animal models of lung fibrosis compared to healthy control animals (Figure 2). Radiolucent areas detected on µCT indicate hyperinflation or emphysema in the lungs [6,41,42]. 

Where in the clinic diagnosis it is still most often a qualitative process based on observation by an experienced radiologist, preclinical research is in essence a quantitative investigation. Visual observations can be quantified based on a scoring system that reflects the extent of lung disease, enabling statistical evaluation of longitudinal changes in the observed lung disease burden [39,41,43]. 

Moreover, µCT enables the quantification of several morphological and anatomical lung biomarkers that describe disease progression and host response ideal for longitudinal follow-up of these diseases [8]. These biomarkers are extracted from a volume of interest covering the lungs thereby avoiding large blood vessels and the heart. Based on a full stacked histogram, a chosen threshold separates the voxels corresponding with radiodense areas from the radiolucent lung voxels, representing respectively the non-aerated lung volume and the aerated lung volume. Moreover, the total lung volume (sum of non-aerated and aerated lung volume, or all the voxels within the lungs) and mean densities within these lung volumes can be derived. Hereby, we can differentiate between pathologies presenting as hyperdense areas (malignancies, infection, infiltrations, consolidation or atelectasis) versus pathologies causing radiolucence on CT such as hyperinflation, air trapping, or emphysema. The non-aerated lung volume is the biomarker that directly quantifies lung disease burden such as lung metastasis, infection, fibrosis and inflammation in animal models [8,38,39,42,44,45,46,47], and would be the direct correlate to other readouts to measure the severity of fibrotic disease such as hydroxyproline assay, or Ashcroft score from histological lung sections [8,9]. 

By applying imaging-derived biomarkers for evaluating models of lung diseases, we were able to highlight previously overlooked aspects of lung diseases, thereby directly affecting our current knowledge and filling the gaps left by investigations that would only employ standard cross-sectional evaluation techniques. One of most important aspects that has previously been overlooked in lung disease research, is that mice often increase their total lung volume to compensate for the increased disease burden and loss in airspaces [8,9,38,44,48,49]. This should be considered as it influences the interpretation of other functional or imaging-derived read-outs, such as the mean lung density or lung volume changes. Where the non-aerated lung volume is a direct quantitative marker for the extent of pathology, the aerated lung volume is regarded as a biomarker of lung function. Indeed, it correlates with volumetric readouts from endpoint lung function measurements such as inspiratory capacity [8,9]. 

In the silica-induced fibrosis model (Figure 2), Dekoster et al. found, contrary to what would be expected for a restrictive disease, an enlarged aerated lung volume during the plateau of the fibrotic phase, consistent with the absence of a lowered inspiratory capacity, FVC, FEV_0.1_ or increased elasticity [9], lung function readouts that would typically be affected in human restrictive disease or in the bleomycin model [6,50]. The key to correct interpretation of lung function readouts lies in the consistently increased total lung volume retrieved from the µCT scans, reflecting enlargement of the lungs with silicosis. We interpret the enlargement of total lung volume, observed in this and other mouse models of lung diseases, as a compensation mechanism for the otherwise excessive loss of airspaces [8,49]. This phenomenon is absent in human patients, but in mouse models this enlargement of the lungs affects the interpretation of µCT- and lung function-derived readouts. Where lung function volumetric readouts may underestimate the presence of restrictive lung disease in this model, µCT offers unambiguous readouts on the presence of inflammation and fibrosis and a potential compensatory reaction thereto by enlargement of (aerated) lung volumes. These findings further emphasize the importance of adding µCT examinations to the standard preclinical workflow to characterize the response of mouse models to lung insults. 

Next to extracting biomarkers of lung disease and host response thereto, µCT offers the additional capability/has an enormous potential for extracting functional biomarkers that reflect lung volumes and function in small animals. There are several options to extract functional biomarkers from lung µCT data. Certain dedicated µCT scanners allow the imaging of ventilated mice, controlled by directly connecting a scanner and a lung function measurement device and using a perturbation called “breath-hold”. With this perturbation, a known volume of air is placed into the lungs for a short period of time (seconds), which allows the µCT to take several projections. To image the complete lungs, this perturbation needs to be repeated several times to result in a high-resolution lung scan. Apart from this perturbation, lung function measures may include forced oscillation lung function measurements that will result in specific tissue and airway related parameters, such as tissue elastance and airway resistance. This setup would allow direct comparison of lung physiology with parameters derived from imaging. On the other hand, we can use µCT as a method to derive functional lung biomarkers from free-breathing mice. Using respiratory gated protocols, µCT can deliver 4D data, meaning that several reconstructions cover different phases of the breathing cycle. This 4D µCT approach allows the extraction of additional biomarkers reflecting vital lung function, such as aerated lung volume and tidal volume, from free-breathing, live mice, thereby complementing data from state-of-the-art lung function measurements. These functional µCT-derived biomarkers can be derived in a longitudinal manner and can better reflect the physiological situation since no forced breathing or intubation is required compared to a ventilated approach. With µCT, it becomes possible to extract longitudinal data on certain aspects of lung function from the same animal without any risk of animal mortality. 

The capabilities of µCT to provide longitudinal, quantifiable visual and biomarker data on different lung pathology aspects make µCT an efficient technology, ready for routine use in lung disease research. µCT can also complement the ample functional information that end-stage lung function measurements provide, while adding regional information on lung pathology, thereby establishing and increasing the synergism of both technologies for routine evaluation of lung conditions and their therapy. 

#### 3.2.2. Radiation Safety of Repeated Low-Dose µCT of Mouse Models of Lung Disease

In spite of the higher x-ray dose the animal is exposed to because of the higher resolution and sensitivity needed to provide a good quality mouse lung µCT-scan of an animal that is about 1000 times smaller than a person, weekly repeated µCT remains without radiotoxic side effects to the lungs of healthy mice [7]. Nevertheless, any remaining concerns related to potential radiotoxicity of the cumulative radiation dose to diseased animals need to be ruled out before we can safely implement µCT for longitudinal studies. The ionizing radiation in µCT acts on biological tissue through two main effects: 1/ the x-rays interact with water molecules resulting in reactive oxygen species and free radicals and 2/ x-rays disrupt the chemical bonds of several biomolecules (nucleic acids, proteins, lipids etc.) [51,52] For example, when x-rays interact with genetic material, they create DNA base modifications, base damage or double-stranded DNA breaks. To assess the biological effects of radiation, not only the cumulative dose of radiation is important, but also the factors that determine this, such as dose-rate, scan frequency and type of exposed tissue. Depending on the intrinsic repair capacity, tissues exposed to the relatively low radiation dose of µCT are able to repair the damage. 

A remaining hurdle towards routinely implementing longitudinal µCT for lung research would be that due to hardware restrictions, it cannot be excluded that the relatively high x-ray exposure administered during the current available high-resolution µCT protocol may still have an effect on the specific aspects of the disease model, such as a possibly altered immune response or alterations in the tissue remodeling processes that are specific to the pathogenesis of lung fibrosis. The assurance of low, harmless dose exposure is extremely relevant in lung disease models that involve rapidly dividing cells, such as the metastatic, tissue remodeling and inflammatory processes investigated in lung research. 

An important step thereto has been taken by investigating the potential effects of repeated x-ray exposure during µCT scanning of animal models of lung metastasis and fibrosis (as models involving rapidly dividing cells may be particularly sensitive to radiation), including potentially altered relevant readouts such as immune cell proliferation and apoptosis. Berghen et al. found a disease-independent small but consistent decrease in circulating platelet and lymphocytes after a weekly repeated 4D respiratory-gated scan for five weeks [53]. Based on these observations, they established an upper safety limit of 540–699 mGy/scan to be delivered on a weekly basis five times with repeated µCT. This limit can be seen as physiologically safe but with a sub-clinical drop in certain blood cell counts. These effects can be completely eliminated by using less-demanding µCT protocols, such as by optimizing 3D imaging protocols and reducing the dose delivered to the mice with each scan [53]. Importantly, the conclusion is that µCT protocols can be designed that do not interfere with radiosensitive processes in the body. Radiation safety is a necessary step for the introduction of µCT into routine preclinical practice, enabling lung researchers to benefit from all its advantages without concerns for radiotoxicity, to benefit the direct translation of imaging examination results from mouse to patients and back. 

#### 3.2.3. Dose Reduction Approaches for Respiratory-Gated µCT

**Figure 3:**
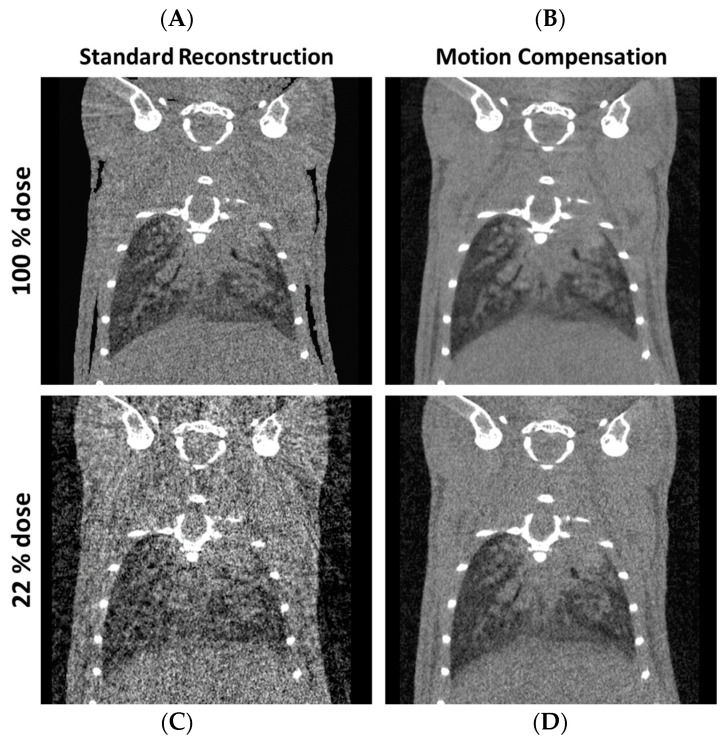
Motion correction of in vivo µCT to improve image quality and dose exposure. Respiratory-gated 4D µCT reconstructions of a mouse with a respiratory window width of 20%. The top row shows reconstructions obtained with the full dose of the used reference protocol (**A**,**B**) and the bottom row shows reconstructions obtained using only 20% of the reference dose (**C**,**D**). The standard reconstruction (FBP) results in severe artifacts if dose is reduced while a motion compensation (MoCo) approach results in an image quality sufficient for most qualitative and quantitative tasks (**D**). (C = 50 HU, W = 400 HU). Videos are available as a supplementary: Video S1a and S1b.

In recent years, a variety of methods have been proposed to decrease the radiation dose administered in preclinical in vivo lung examinations. These methods comprise improvements in system hardware and image reconstruction algorithms alike. In early experiments, time-resolved volumes were reconstructed using filtered back projection (FBP); i.e., x-ray projections were continuously acquired alongside a corresponding respiratory signal and respiratory-gated volumes were retrospectively obtained by reconstructing only those projections measured in a desired motion state (respiration phase). To reconstruct the entire respiratory cycle, i.e., to obtain 4D data consisting of 3D volumes at different time points, several reconstructions have to be performed, preferably with overlapping phases. Since only a fraction of all acquired data is used for the reconstruction of a single volume, image quality is usually degraded by streak artifacts unless vast amounts of projections are available, eventually increasing radiation dose [54,55,56,57]. Ultra-high-resolution in vivo acquisitions of the lung might also require additional cardiac-gating since uncompensated cardiac motion impairs image quality and reduces spatial resolution due to motion artifacts. In turn, even more projections are required to maintain a sufficient image quality in case of simultaneous cardiac- and respiratory-gating, adding to the administered radiation dose. If only a single respiratory motion state is desired, prospective gating might be used; i.e., projections are only acquired if the lung is in a particular motion state. This, however, requires dedicated hardware that is either difficult to handle or might not be readily available in practice, e.g., ventilators with a feedback-loop to the micro-CT scanner or pulsed x-ray sources [58,59]. Dedicated iterative reconstruction methods have proven to reduce the administered radiation dose by an order of magnitude while maintaining the image quality and accuracy of derived quantitative measures [60,61,62]. In brief, these methods incorporate prior knowledge into the image reconstruction process identifying and removing noise and artifacts. For example, one might assume that the CT-values of the specimen under investigation are piecewise constant or that adjacent breathing phases show similar anatomical structures. An even higher radiation dose reduction is promised by motion compensation (MoCo) methods that have initially been developed for image-guided radiation therapy and have been translated to clinical PET, clinical MRI and preclinical imaging later on [63,64,65,66,67]. In particular, given a series of respiratory-gated FBP reconstructions covering the complete respiratory cycle, these methods estimate motion vector fields between adjacent respiratory phases and ensure that these vector fields are cyclic. As soon as all motion vector fields are available, the initial reconstructions are deformed and superimposed to match a desired respiratory phase. Since all acquired data contribute to each final image, this process is much more dose-efficient compared to previous algorithms (Figure 3, Video S1a and S1b in the Supplementary Materials). However, one should note that the computational demands for iterative reconstructions or MoCo methods are higher compared to conventional FBP reconstructions. While an FBP reconstruction can be performed within seconds using modern hardware, algorithms that are more sophisticated might exhibit runtimes in the order of minutes or hours. Hence, conventional respiratory-gated FBP reconstructions are available in almost all commercial micro-CT systems while other methods, though promising, are only adopted slowly into preclinical practice.

#### 3.2.4. In Vivo MRI for Small Animal Lung Fibrosis Imaging

The rationale for developing MRI as a complementary imaging option is because µCT lacks the sensitivity to distinguish inflammatory from tissue remodeling processes, a concept that is very relevant for the pathogenesis of lung diseases, particularly in lung fibrosis. MRI is a more versatile tool that in theory could enable monitoring of different cellular parameters and contrast mechanisms, providing complementary information to µCT on different pathogenic processes in the lung, ultimately aiming at discriminating between inflammatory and lung remodeling processes in vivo. A model of particular interest in the exploration of the potential of MRI to distinguish between inflammation and fibrosis is (bleomycin-induced) lung fibrosis, as this disease involves both very well characterized initial inflammation and progressive fibrosis. MRI developments, in line with previous work [5], are directed towards designing advanced lung MRI and data processing methods that will enable to unravel inflammation from lung fibrosis and to detect early onset, followed by mild pathology, ultimately aiming at improving the understanding and treatment of pulmonary fibrosis in relevant animal models and towards translation in patients. This is relevant for screening treatment options that can truly halt or even reverse lung fibrosis. 

However, MRI of the lungs is not obvious. Lungs are mainly filled with air, which results in a very low inherent signal that is available for lung imaging. Moreover, the many tissue–air interfaces result in magnetic susceptibility effects that may cause imaging artifacts and accelerate signal decay (short T2/T2*). Therefore, conventional MRI protocols result in little or no signal so that lung tissue appears dark on MR images. Nonetheless, the zero signal in the lungs can provide a useful background against which to recognize abnormalities that produce an increase in signal, such as lung fibrosis or cellular infiltration [68,69,70,71,72]. Nevertheless, the susceptibility effects at tissue–air interfaces are likely to hinder the early detection of smaller fibrotic patches or sites of disease onset at earlier stages of pathogenesis when using conventional MRI protocols. To overcome these limitations, we can use a pulse sequence allowing for ultra-short echo times (UTE) [73]. In UTE MRI, image contrast is less influenced by air–tissue susceptibility effects, and the detection of tissues that particularly suffer from these effects, such as lung and fibrotic tissue, therefore improves significantly compared to conventional pulse sequences [5]. UTE MRI is currently under evaluation for clinical applications [74,75], but was only recently introduced for mouse lung imaging [76,77,78]. We have shown that its potential in evaluating lung fibrosis development is promising [5], but its ability to discriminate inflammatory from tissue-remodeling processes remains under-evaluated. 

UTE-MRI can potentially monitor inflammatory events using established approaches that utilize iron oxide-based nanoparticles [79,80]. Alternatively, ^19^F-MRI can be used to visualize the accumulation of immune cells in the lungs with disease progression. In contrast to conventional proton-based MRI, ^19^F-MRI can visualize cells labeled with ^19^F-based contrast agents without a misleading background. Hereby, the visualization of immune cells after systemic administration of ^19^F-based agents has already been established [81,82]. Detection of lung pathology may suffer from movement artifacts caused by breathing and the beating heart, unless the acquisition is triggered to this motion. Motion can be monitored based on the MRI signal intensity variations induced by cardiac and respiratory movements [83], a technique known as “self-gated” MRI. This technique obviates the need for respiration pads and ECG leads, thereby enhancing the throughput in animal studies. The self-gated MRI technique has been introduced for mouse cardiac and rat liver imaging [84,85,86], and for (mouse) lung MRI [5]. 

MRI developments that will enable us to identify the switch from inflammatory to lung fibrosis will greatly improve our understanding and treatment of pulmonary fibrosis and is fully translational. Although further development is needed before MRI can be implemented as such, this is relevant for screening treatment options that can truly halt or even reverse lung fibrosis as these would improve the merely symptomatic or anti-inflammatory treatment options that are currently available [2].

#### 3.2.5. In Vivo Optical Imaging of Lung Diseases: From the Whole-Body Level to the Cellular Scale 

Whereas µCT enables the visualization of inflammation and fibrosis onset and progression on the level of the whole lung, optical imaging approaches are currently underused, but hold the promising potential to provide complementary information on the presence of certain biomarkers of disease at a whole-body, whole-organ and/or microscopic level. 

On the whole-body level, bioluminescence or fluorescence imaging of cell populations labeled with genetically expressed reporter genes is available to track pathogens during lung infection, metastasis or inflammatory processes in small rodents [4,38,45,87,88]. With specific fluorescent dyes, tagging a certain cell, protein or responsive to a certain enzymatic marker relevant to the disease process, certain contributions from the immune response to fibrosing disease development can be non-invasively monitored with optical imaging [89]. When using dyes, these probes have translational potential for application to human samples such as the BAL fluid. Similar to radionuclide-based imaging approaches such as PET or SPECT, the success of any probe or tracer-based approach to be a diagnostic or prognostic biomarker depends on several factors such as biodistribution, but most importantly on the specificity of a certain tracer for a particular cell or protein and thorough validation of its role in the disease process [37]. 

It currently remains a challenge to observe in real time and repeatedly the dynamics of cellular interactions in the lung at a sufficiently high resolution. Intravital microscopy (IVM) approaches enable the observation of cellular processes in the lung in real time at microscopic resolution, which may lead to novel observations and insights in the dynamics of pathogenic processes and interactions with the host. This requires direct access to the lungs. One way to achieve this is by introducing an optical window in the chest wall to contact the lung. This has been technically achieved and allowed real-time investigation of lung microvasculature or inflammation in the context of metastasis or ILD with single-cell resolution up to several hours, but remains a terminal procedure due to its invasiveness [90,91,92,93,94,95]. 

Another option to access the lung is the use of IVM by fibered confocal fluorescence microscopy (FCFM) via an endoscopic procedure as an innovative, complementary optical imaging technique to visualize processes in the lung in real time and at microscopic resolution [96]. By rational use of activatable and/or environment-sensitive fluorescent probes specific to a certain cell or molecule, real-time fluorescence microscopy inside the lungs of a free-breathing, anesthetized mouse comes within reach [91,97,98,99,100,101,102]; e.g., probes for targeted imaging of subpopulations of macrophages, and probes to image cathepsin activity, which may be relevant in cystic fibrosis and fibrosing lung disease. Thereby, we will be able to visualize specific processes such as the extent of inflammation in vivo at different time points in the same animal, in interaction with other processes based on labeling with green and red fluorescent probes/dyes. IVM technology by FCFM can be regarded as an addition to the available imaging technology platform for the preclinical evaluation of specific contrast agents targeting molecular and/ or cellular biomarkers of certain stages of disease. This has a direct potential for clinical translation for improved differential diagnosis, in the clinical context better known by its name “probe-based confocal laser endomicroscopy” (pCLE) [103]. 

### 3.3. Non-Destructive 3D Ex Vivo Lung Imaging

As an alternative for the current 2D ex vivo validation options, 3D examination of the intact organ would result in a better quantifiable and comprehensive analysis method, specifically relevant to lung research because of the need to evaluate the lungs as a whole in 3D. Sample selection for ex vivo examination can be done at relevant time points during pathogenesis as defined by in vivo imaging. Thereby, a technological gap is bridged by providing the missing link between whole-body imaging methods that visualize overall anatomy and disease in 3D, and microscopic techniques that give information with high, cellular resolution in 2D or with limited penetration depth and with a very small field-of-view, such as the aforementioned IVM approaches. Such technology enables us to complement in vivo findings with a more detailed analysis in the context of the intact organ.

#### 3.3.1. Ex Vivo µCT Visualizes Lung Parenchyma and Vasculature in 3D

**Figure 4:**
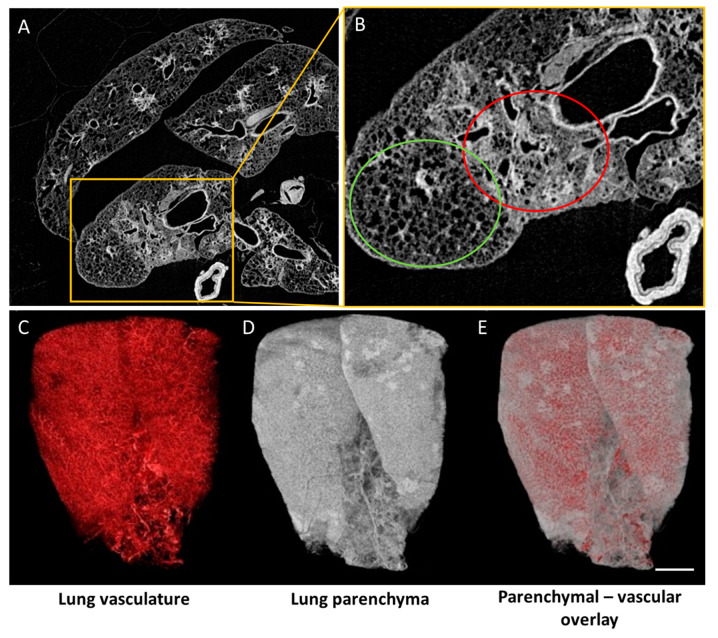
High resolution ex vivo µCT of isolated rodent lung. (**A**) Reconstructed µCT of whole mouse lung, visualizing spatial differences in lung pathology; (**B**) enlargement of the yellow box in A, zooming in on spatial differences in areas with localized lung inflammation (red circle) and non-pathological regions (green circle); (**C**–**E**) Ex vivo µCT of rat lung with barium-based vascular perfusion. Vascular network rendered with maximum intensity projection (MIP) (**C**); lung parenchyma visualized using minimum intensity projection (MinIP) (**D**); an overlay of parenchymal and vascular tissue (**E**). Scale bar 5 mm. Video is available as Supplementary Video S2.

Ex vivo µCT is a significant step in correlating in vivo µCT and ex vivo histological examination of diseased lung tissue for comprehensive preclinical research. It is one of few methods providing 3D imaging of the whole extracted organ with isotropic micrometer resolution (Figure 4, Video S2 in the Supplementary Materials). Moreover, this modality, combined with contrast agent perfusion or staining methods, produces images that can compete with classical histological sections [104,105,106]. µCT allows fast automated analysis of hundreds of cross-sectional images separated by a few microns. Any attempt to reach equivalent results using histological methods would be a tedious, time consuming, and most importantly, a sample-destructive process. 

µCT-captured datasets enable advanced 3D analysis, modeling and internal structure assessment of the organ as well as interrelations between its subcomponents. Specified perfusion and/or staining provides images of chosen tissues, for instance, vascular networks (Figure 4C, Video S2 in the Supplementary Materials). High resolution ex vivo µCT methods enable qualitative and quantitative characterization of the vascular network of the lungs and analysis of morphometric parameters like volume, surface, and local diameter of the vessels [107], along with evaluation of complexity of the lung circulation system using, for instance, connectivity density or fractal dimension.

Lung vasculature high resolution ex vivo µCT imaging is invaluable in research on pathophysiology of numerous lung illnesses that often reduce patients’ quality of life by causing shortness of breath and chest pain. Group of disorders known as pulmonary vascular disease (PVD), such as pulmonary arterial hypertension or pulmonary embolism, significantly affect blood flow in the pulmonary circulation system resulting in severe disability or death [108], warranting towards the important vascular component in lung disorders. High resolution ex vivo µCT can visualize pathological angiogenesis, present in chronic thromboembolism, asthma, cystic fibrosis and especially in primary tumors [109,110]. 

The µCT results are not direct. During the measurement, the detector records hundreds of 2D projections, which are then subjected to an image reconstruction process, as a result of which we obtain cross-sectional images of the scanned object. Optimization of image reconstruction parameters, as well as the modification of transfer functions when viewing the 3D model, provide an opportunity to zoom in and observe extremely small and different details based on the same scan.

#### 3.3.2. Optical Imaging of Intact Lungs with OPT and SPIM

Next to ex vivo µCT, optical projection tomography (OPT) [111] and selective plane illumination microscopy (SPIM) [112] of optically cleared mouse lungs can be a non-destructive alternative for the ex vivo validation options that involve mainly histological sampling of mouse lungs in 2D. OPT can be thought of as an implementation of the principles of CT, but in the visible region of the spectrum. SPIM is a technique in which optical sectioning is achieved directly, by exciting fluorescence with a thin sheet of laser light. OPT and SPIM are novel meso- and microscopic approaches that enable us to validate and complement in vivo imaging results and to study the spatial distribution of lung pathology in intact mouse lungs in 3D with cellular resolution [111,113]. As OPT and SPIM are optical imaging modalities, we have the possibility to exploit different fluorescence channels, thereby opening the door towards multiplexing of different signals from differentially labelled cells. 

Given the size and optical properties of adult mouse lungs, imaging them in toto in their native state is not practical. To avoid the tedious and destructive process of physically sectioning the lungs, several methods have been proposed to chemically clear tissues and organs to make them optically transparent. These include organic solvent-based techniques such as treatment with benzyl alcohol + benzyl benzoate (BABB) [111] or ethyl cinnamate [114], aqueous methods such as the CUBIC family of protocols [115], or hydrogel-based protocols such as CLARITY [116] or expansion microscopy [117]. Each method has its advantages and disadvantages in terms of clearing quality, fluorescence preservation, toxicity of reagents, and time required; see Ueda et al. [118] for a recent review.

The combination of OPT and/or SPIM with chemical clearing of intact murine lungs is an important addition to the technological toolbox for the study of lung diseases. Although it is an ex vivo technique, and therefore limited to end-point analyses, it can add significant value. The enormous variety of molecularly specific labels (fluorescent proteins, fluorophore-labelled antibodies, small molecular dyes) available for optical imaging mean that the 3D distribution of specific cell types or cells in particular states can be visualized. The fact that the entire, intact lung can be imaged permits the detection of rare cells or events that would likely be missed if only sub-regions of the lung are examined.

Other than simply providing “additional data”, the datasets generated by OPT and SPIM of ex vivo, cleared lungs have two main uses. Firstly, they can be correlated with previous in vivo data, to confirm observations and re-examine regions using new contrasts. Secondly, they can be used to guide future physical sectioning for traditional 2D histology of lung slices, if required. This is possible because at least some of the clearing protocols (e.g., treatment with BABB) are reversible; that is, a lung can be chemically cleared, imaged in 3D, and then returned to its initial state by inverting the clearing protocol [119].

**Figure 5:**
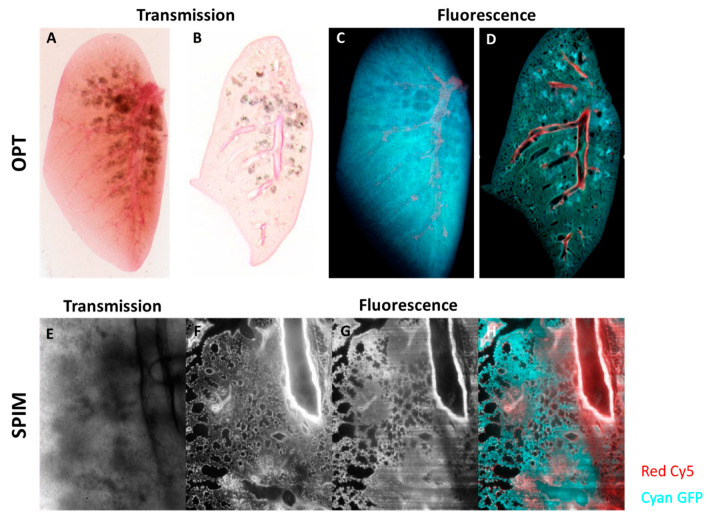
Optical Projection Tomography (OPT) and Selective Plane Illumination Microscopy (SPIM) of silica-induced lung fibrosis. Mice were oropharyngeally instilled with crystalline silica particles (5 mg/mice), sacrificed and imaged 35 days after instillation. OPT transmission imaging of optically cleared lungs shown as a “raw” projection (**A**) and as a reconstructed slice (**B**). The regions with concentrations of silica particles can be seen as grey blotches in the transmission images (**A**,**B**). OPT fluorescent images of projected (**C**) and reconstructed (**D**) lungs. In fluorescence, the larger vascular structures can be seen in the Cy5 channel (red in images **C**,**D**), and the general structure is visualized using autofluorescence in the GFP channel (cyan in **C**,**D**). A more in-depth and spatial analysis of optically cleared lungs using SPIM shows a transmission image (**E**) and corresponding fluorescence optical slices in the Cy5 (**F**) and GFP (**G**) channels. (**E**) is a transmission image taken in the SPIM showing a field of view with regions containing silica particles (note that this is a traditional wide-field image, not an optical section). Optical sections of fluorescence are shown in (**F**–**H**), where regions with both normal structure and fibrosis can be seen. The images in (**F**,**G**) are shown in color overlay in (**H**). 3D visualizations of the OPT can be found as Supplementary Videos S3a and S3b.

We have been able to successfully demonstrate the possibility to optically clear large samples such as adult mouse lungs for compatibility with OPT and SPIM [120] (Figure 5, Video S3a and S3b in the Supplementary Materials). For this we generally use the BABB protocol, for its simplicity, speed, and quality of clearing. Figure 5 shows a cleared mouse lung imaged using both OPT (A–D) and SPIM (E–H), demonstrating its power for non-destructive optical sectioning of inflamed and fibrotic lungs based on autofluorescence of tissue components. The OPT and SPIM-approach benefit the experimental workflow to provide regional information on lung pathology that can be used for identification of areas of interest for subsequent classical histology on the same lung samples for direct correlation. 

## 4. Discussion 

### 4.1. A Multimodal Lung Imaging Approach to Improve Translation between Bench and Bedside

Despite the high burden of disease, effective anti-fibrotic treatments for many (idiopatic) lung fibrosis patients are not yet available. This is surprising, as many pathways have been successfully challenged in preclinical models, with exponential growth in studies and compounds but limited translation towards the clinic. Some of these failures might be attributed to the relatively poor performance of the preclinical models and in particular the use of end-stage analyses that likely reflect the sum of disease activity over time more than the extent of the disease process itself. Most of the preclinical models are self-limiting, thereby potentially overestimating the effect size of any intervention. The high costs associated with the model set-ups and the laborious pathology analysis also leads to a relative lack of replicated experiments. 

The availability of novel diagnostic technologies now challenges the current gold standard and seriously questions the methodological dogmas in preclinical lung research, and suggests that these issues can be overcome by the development of imaging methods that catch the dynamics of the model and therefore can assess disease activity and outcome over time, and that can potentially replace the need for full pathology analysis in screening and replication set-ups. Because of the disappointing results in translating mouse model results to the clinic [1,2], many questions remain in particular related to the choice of the optimal biomarker(s) to address a specific research question, evaluate mouse models for their validity and experimental therapies for translational studies. 

Biomarkers that can be derived from in vivo imaging have proved to be relevant for the in vivo evaluation of lung fibrosis and its therapy, and the host response thereto [8,49]. Based on the finding that mouse lungs expand significantly during lung fibrosis progression—a phenomenon that remains largely overlooked—it proves to be essential that lung fibrosis and its therapy are evaluated in 3D and over the entire lung [8,9,49]. This is in contrast to the current preclinical gold standard of 2D histological sampling methods, which overlook potential compensatory mechanisms in the mouse lung and therefore may underestimate the true extent of pathology. Adding µCT-derived biomarkers throughout lung disease and therapy investigations should become part of the new standard diagnostic methods in experimental lung disease investigations. Better evaluation of therapy results in available and novel rodent models of lung disease will be essential in resolving the issues with translation of results from bench to bedside. The example from the chronic lung fibrosis model showcased here indicates that direct translation from the clinical view on obstructive and restrictive diseases is not so straightforward in these animal models and more importantly, it demonstrates that in vivo µCT is essential for the correct interpretation of preclinical lung research [9]. We expect a non-invasive imaging approach to enable us to identify and resolve at least some of the anomalies encountered when translating apparent successful preclinical therapy trials to patients. 

To have a strong contribution to the mechanistic knowledge about pulmonary diseases and co-morbidities and towards improving disease management, we need complementary and comprehensive approaches. By innovating diagnostic technologies as outlined, we can push the state-of-the-art forward and break new ground. Improving and applying imaging technologies may lead to novel observations and new insights. A combination of multiple state-of-the-art and innovative in vivo and ex vivo imaging modalities and techniques such as (µ)CT, lung MRI and MoCo, intravital microscopy, high-resolution ex vivo µCT, OPT and SPIM can ultimately overcome limitations of the individual techniques and provide complementary information. 

### 4.2. Ethical Impact and Considerations of Non-Invasive Imaging in Lung Research

Given the type of research, there is no alternative to the use of animals for studying the complex interplay of host immune cells and triggers of pathology in the different processes that are characteristic of pulmonary diseases. Nowadays a hot topic, and an always important societal challenge that the lung research community faces, is how we deal with the ethical burden related to research involving live animals. The acceptability of using animals in research rests on the twin expectations that the research findings will be meaningful and substantial, and that the suffering will be minimal. For scientific justification, the validity of science is dependent on the validity of the animal model; that is, dependent upon the extent to which experimental findings in the animal model can be generalized to other species, particularly humans. The ethical justification is based upon the view that increasing the knowledge base makes it possible to develop therapies that mitigate pain and suffering caused by illness and trauma, and thereby responds to the moral imperative to do well. It assumes that research can be carried out with no or minimal discomfort or distress to the animal research subjects, and that any pain and suffering experience is compensated for by the alleviation of human pain and suffering caused by disease and injury. The focused procedures and efforts of a non-invasive imaging approach answer to both the scientific and ethical justification. Such an approach will directly lead to a reduction and refinement in the use of animal models in biomedical research because of the strong focus on the development, optimization and use of non-invasive imaging methods and will diminish significantly the “harm-to-benefit” ratio in the justification of animal use in this area of research. Innovation in imaging will immediately be able to suggest generic non-invasive imaging protocols for the broad lung research field, yielding a more accurate evaluation of animal models relevant to human disease. Imaging allows us to longitudinally monitor individual animals, thereby extracting meaningful biomarkers before the onset of signs of distress, greatly reducing the number of animals that are typically used in these kinds of studies. 

Most, if not all, of the multi-modal imaging approaches showcased here are generic and will have important impacts on the assessment of lung diseases in general (e.g., infection models and lung tumor/metastasis models, cystic fibrosis models, COPD and asthma models, and other (transgenic) lung fibrosis models). 

## 5. Conclusions

Whereas clinical practice is unthinkable without imaging for disease evaluation, in the preclinical field the available imaging technology is still most often not employed in spite of its obvious advantages. More interdisciplinary collaboration as described in this showcase may change this and may bring clinicians, lung and imaging research communities closer together, to interact and integrate more for the benefit of the research community, the animals used in the studies, and patients. We strongly believe that a multifaceted approach to lung disorders, including cooperation between those doing basic research and clinical studies, will improve translation, our understanding of the disease and, above all, provide benefits for our patients. Our imaging approach will therefore not only impact on academic research, but also on business development through industry collaborations extending into pharma companies that may change their study design regarding drug testing towards implementation of a more imaging-based approach. 

## Supplementary Materials

The following are available online at https://www.mdpi.com/2075-4418/10/9/636/s1, Video S1a: no MoCo, Video S1b: MoCo. Video S2: ex vivo lung µCT, Video S3a: transmission OPT, Video S3b: fluorescence OPT.
