# Supplementary figures and images for: From Mouse to Man and Back: Closing the Correlation Gap between Imaging and Histopathology for Lung Diseases
Source: Diagnostics (Basel). 2020 Aug 26;10(9):636. doi: 10.3390/diagnostics10090636 (PMC7554749; doi:10.3390/diagnostics10090636)

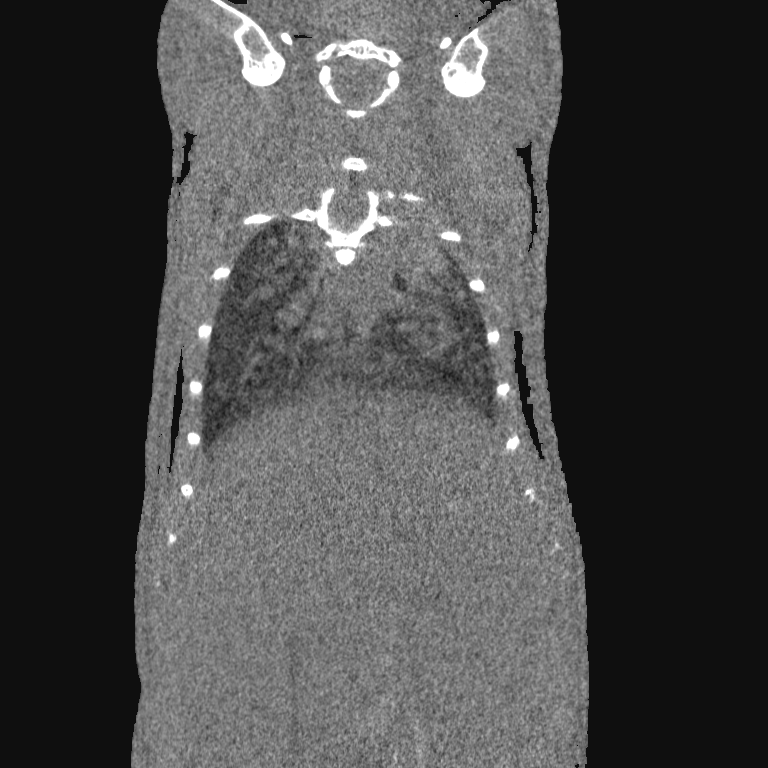

Supplement: Supplementary file 1 [file diagnostics-10-00636-s001.zip › Supplemental video 1a no MoCo.gif]

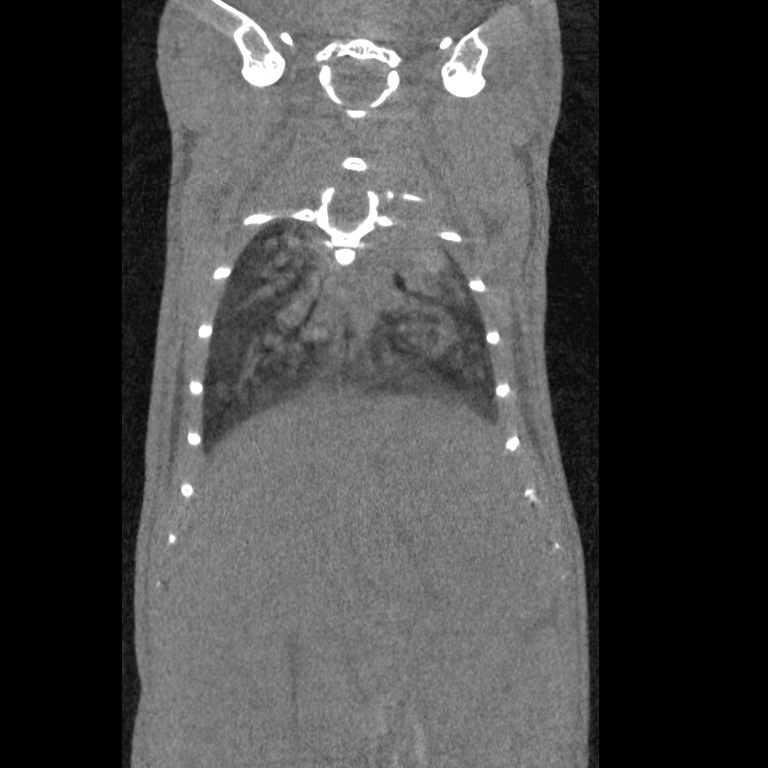

Supplement: Supplementary file 1 [file diagnostics-10-00636-s001.zip › Supplemental video 1b MoCo.gif]
